# Who Are the Stalkers in Hong Kong? Examining Stalking Perpetration Behaviors and Motives of Young Adults

**DOI:** 10.3390/ijerph182312798

**Published:** 2021-12-04

**Authors:** Heng Choon (Oliver) Chan

**Affiliations:** Teaching Laboratory for Forensics and Criminology, Department of Social and Behavioral Sciences, City University of Hong Kong, Hong Kong, China; oliverchan.ss@cityu.edu.hk; Tel.: +852-34429223

**Keywords:** stalking, perpetration, stalker, stalking behavior, stalking motive, Hong Kong

## Abstract

Information on the stalking perpetration dynamics of young male and female adults in Asian countries is scarce, particularly in relation to stalkers’ offending characteristics, perpetration behaviors, motives, and other violent and nonviolent behaviors. This study compares the stalking perpetration dynamics (i.e., offending characteristics, lifetime stalking perpetration behaviors and motives, and other violent and nonviolent behaviors) of young male and female adults in Hong Kong. Of the 2496 participants, recruited from all eight public and two private universities in Hong Kong, 45 participants (1.8%; mean age = 22.84 years) reported stalking perpetration during their lifetimes (33 males (mean age = 22.56 years) and 12 females (mean age = 23.58 years)). Significantly more males than females reported that they had engaged in stalking perpetration in the past 12 months. In general, participants most frequently perpetrated surveillance-oriented stalking behaviors, followed by approach-oriented stalking behaviors and intimidation- and aggression-oriented stalking behaviors. Significantly more females than males reported to have threatened to harm or kill their victims. Additionally, significantly more females than males reported “the victim caught me doing something” as their motive for stalking. The findings of our study provide useful information for prioritization during criminal investigations. Increased understanding of the stalking perpetration dynamics of males and females will help the police and threat assessment professionals to formulate their investigation and management plans.

## 1. Introduction

Stalking is a global public health issue that impacts many people each year. Large-scale representative surveys conducted in Australia, the U.S., and the U.K. during the past decade largely identified similar lifetime prevalence rates of stalking victimization, with one in five women and one in 12 men in Australia and the U.K. [1,2] and one in six women and one in 18 men in the U.S. experiencing stalking victimization [3]. There are various definitions of stalking, from a narrow legal definition that requires the stalker to demonstrate intent and the victim to feel fear, to broader definitions that encompass lists of constituent behaviors (see [4]). Although most of the research on stalking has been conducted in Australia, the U.S., and the U.K., an increasing number of studies conducted in under-researched populations (e.g., Denmark, Finland, Germany, Ghana, Hong Kong, Lithuania, mainland China, the Netherlands, Portugal, South Africa, Singapore, and Spain) have found stalking to be a common, and perhaps universal, problem [5,6,7,8,9]. The traditional view of stalking is that the perpetrators of stalking are more likely to be male, whereas the victims are more likely to be female [10]. Indeed, a meta-analysis by Spitzberg [11] indicated that over 70% of stalkers were male, and more than 80% of victims were female. A further meta-analysis of 175 studies by Spitzberg and Cupach [4] concluded that females were more likely than males to experience stalking victimization at some time in their lives (28.5% vs. 11% lifetime risk, respectively). Only a few studies in Spitzberg’s [11] review reported stalking perpetration rates, and of these, the mean rates were 16% for males and 9% for females.

Stalking may consist of a wide array of behaviors, ranging from harassment (e.g., standing outside the victim’s home, showing up to a victim’s location) to life-threatening behaviors (e.g., threats to harm or kill the victim) [6,12]. Studies have identified the most frequent perpetrators of stalking to be ex-intimate partners (49–81%), followed by acquaintances (13–22.5%) and strangers (10–18%) [11,13,14,15]. Most ex-intimate partner stalkers appear to be motivated by the need to control their victim or the desire to restart a relationship [16,17]. Other common motives include a desire for sex, seeking revenge, and victim intimidation [4]. A victim’s risk of experiencing psychological, social, and physical harm increases the longer they are subjected to stalking behaviors and the types of coping strategy adopted [18]. Rejected ex-partners and prior acquaintances have been found to be the most persistent stalkers, while strangers are the least persistent [19]. A stalking victim is also at the greatest risk of physical violence when the stalker is an ex-intimate partner [20].

As stalking is largely viewed as a gendered offense, most research on stalking perpetration has focused on male stalkers. Only a small number of empirical studies have examined female stalking perpetration, e.g., [21,22,23,24,25,26]. The majority of these studies reported comparable rates of violence for male and female stalkers [21,23,27], although female stalkers seem to be more persistent than male stalkers [19]. However, the type and severity of male- and female-perpetrated violence has not yet been compared in detail. Preliminary research has reported that female stalkers are primarily motivated by the desire to establish an intimate relationship with their victims, whereas male stalkers are more likely to seek to maintain a relationship with a former intimate partner [21,23]. Female stalkers are less likely than male stalkers to target strangers and are more likely to engage in same-sex stalking perpetration [21,23].

Hong Kong, geographically situated in the Asia Pacific region, has been a special administrative region (SAR) of the People’s Republic of China (PRC) since July 1997. Before its return to the PRC, Hong Kong was a British colony for more than 150 years. It is noteworthy that stalking has yet to be legislated against in Hong Kong. Although the Hong Kong Law Reform Commission (LRC) published a report on stalking in 2000 [28], the devastating nature of stalking failed to attract much public attention until the Hong Kong Government [29] published a consultation paper to consult the public on an anti-stalking law in December 2011. Subsequent to the consultation period ending in March 2012, the Government commissioned a consultant to study the experience of overseas jurisdictions in enacting anti-stalking legislation, and the findings were presented to the Legislative Council Panel on Constitutional Affairs in December 2013 [30]. However, nothing has since been announced by the Government about this potential legislation.

Against this background, this study is particularly important for two reasons. It is the first empirical study to compare the detailed stalking perpetration dynamics (i.e., offending characteristics, lifetime stalking perpetration behaviors and motives, and other violent and nonviolent behaviors) of male and female stalkers. This study also adds geographical diversity to the literature on stalking, as it draws from a large sample in an under-researched population: Hong Kong-based male and female young adults. Exploring stalking perpetration is important to identify misconceptions that the general public may hold about stalking behavior and its severity, and appropriate responses to it. If left unaddressed, misconceptions may lead to a lack of demand for policy and social change [6,31]. This is utterly critical in jurisdictions that have yet to introduce anti-stalking related laws. It is important to note that stalking has yet to be legislated against in Hong Kong. Hence, in addition to advancing our knowledge on the topic, the findings of this study may provide insights to inform practice in relation to offender rehabilitation, and development or refinement of public and social policies to help curb the phenomenon of stalking perpetration. It is hypothesized that the offender’s sex will influence the stalking perpetrators’ behaviors and motives, and their other violent and nonviolent behaviors perpetrated against the same victim. In view of the paucity of existing evidence, no directional hypotheses can be stated.

## 2. Methods

### 2.1. Participants and Procedure

#### 2.1.1. Master Sample

The study sample consisted of 2496 participants recruited from the student bodies of eight public (i.e., government funded) and two private universities in Hong Kong. The participants were 56% female (n = 1392) and 44% male (n = 1,104), with a mean age of 21.88 years (*SD* = 3.28). The majority of the participants (about 75%) were approached on the university grounds (e.g., reading corners, common areas, libraries, and student cafeterias), while the remainder were recruited during class breaks or end-of-class sessions with prior consent from the relevant instructors. Ethical approval was obtained from the institutional review board of the first author’s university of employment. The participants’ informed consent was acquired before the paper-and-pen questionnaire was administered. The participants completed the questionnaire in a private area without interruptions and were assured that their responses would be kept confidential and used only for research purposes. Participation in this study was completely voluntary and anonymous, and no monetary incentive was offered. The average time taken to complete the questionnaire was 25 min, and the response and cooperation rate for the survey was around 90%.

The operational definition of stalking adopted in this study was taken from the Stalking Report of the Law Reform Commission of Hong Kong [28]. Participants were first presented with the following definition: “Stalking may be described as a series of acts directed at a specific person that, taken together over a period of time, cause him/her to feel harassed, alarmed or distressed.” For screening purposes, the participants then responded yes or no to questions about whether they had personally experienced and/or perpetrated in stalking under this definition.

#### 2.1.2. Study Sample

A total of 1.8% of the participants (n = 45) reported stalking perpetration during their lifetime: 73.3% were males (n = 33) and 26.7% were females (n = 12). These 45 participants constituted the study sample. The participants’ average age was 22.84 years (*SD* = 3.70; range = 18–36), and there were no significant sex-related differences (males: *M* = 22.56, *SD* = 3.47; females: *M* = 23.58, *SD* = 4.34; *t* = −0.73, *p* > 0.05). The majority of the participants (77.8%, n = 35) were local Hongkongers, and the rest were from mainland China (17.8%, n = 8) or other countries (4.4%, n = 2; Taiwan and France). Nearly two thirds (64.4%, n = 29) were single, three quarters (75.6%, n = 34) reported having no religious affiliation, and 97.8% (n = 44) had obtained at least a secondary school education. Only two participants, both males, reported having been arrested for physical assault; they were aged 17 and 21 years at the time of arrest.

### 2.2. Measures

Self-report measures were created to explore the participants’ offending dynamics (i.e., frequency, duration, stalker–victim relationship, types of and motives for stalking perpetration behaviors, and other violent and nonviolent offending behaviors perpetrated against their victims). The questionnaire containing these measures was printed in both English and Chinese. To ensure the accuracy of the Chinese version, the measures, initially written in English, were translated into Chinese by an experienced and academically qualified English-to-Chinese translator. They were then back-translated into English to ensure face validity and compared with the original English version to confirm consistency.

#### 2.2.1. Stalking Perpetration Behaviors

The participants were surveyed about their experiences with stalking perpetration both over their lifetimes and during the past 12 months. A 12-item scale of the types of stalking behavior perpetration was adopted from Amar’s [32] study: nine items were drawn from the “National Violence Against Women Survey” (NVAWS) (see [14]) and the other three items covered stalking behaviors frequently described in the literature. These 12 stalking perpetration behaviors were categorized into three distinct behavioral themes: (1) surveillance, (2) approach, and (3) intimidation and aggression. The participants were asked to indicate, “Which of the following behavior(s) have you engaged in?” They were allowed to select more than one behavior. Sample items included the following: “Followed or spied” (surveillance item), “Made unsolicited phone calls” (approach item), and “Threatened to harm or kill” (intimidation and aggression item). Cronbach’s α was 0.89 (males = 0.87, females = 0.94).

#### 2.2.2. Stalking Perpetration Motives

To measure the perpetration motives of the participants, the questionnaire included 12 items developed by Baum and colleagues [33] in their representative study of stalking victimization in the U.S. The participants were asked about their reason(s) for engaging in stalking perpetration. They were allowed to select more than one motive. Sample items included the following: “To control the victim,” “To get the victim back into the relationship,” and “Found the victim attractive.”

#### 2.2.3. Other Violent and Nonviolent Perpetration Behaviors

A 10-item measure, developed by Baum and colleagues [33], was used to assess additional violent and nonviolent offending behaviors perpetrated against victims by the participants. Sample items included the following: “hit/slapped/knocked down the victim,” “raped/sexually assaulted the victim”, and “illegally entered the victim’s house/apartment.”

### 2.3. Analytic Strategy

In addition to frequencies, cross-tabular analyses were computed to explore the following: any sex differences in the offending characteristics during the participants’ most recent (i.e., preceding 12 months) perpetration experience; their lifetime experiences with stalking perpetration behaviors and motives; and other violent and nonviolent perpetration behaviors against the same victims. A measure of association (Phi coefficients (between two variables on two levels of each variable)) was conducted to interpret significant findings regarding the strength of the relationships, and, most importantly, to identify *meaningful* patterns. Using Cohen’s standard for the interpretation of the cross-tabular effect size, phi values of 0.29 and under were considered small effects, values between 0.30 and 0.49 were regarded as moderate effects, and values of 0.50 and above were considered strong effects see [34]. Unadjusted odds ratios (ORs) were used to calculate the odds that an outcome (i.e., the stalker’s modus operandi) would occur when a participant was faced with a particular exposure (i.e., the stalker’s intention to engage in particular stalking behavior) compared with the odds of the outcome occurring in the absence of that exposure.

## 3. Results

### 3.1. Offending Characteristics of the Most Recent Stalking Perpetration

Table 1 presents one significant sex-related difference in offending characteristics. Although 41.9% of the participants had engaged in stalking perpetration in the past 12 months, this figure included a significantly larger proportion of males than females (50% males vs. 18.2% females; χ^2^ = 3.41, *p* = 0.049). The strength of this relationship approached a moderate effect size (Phi = 0.28). Nearly half (48.8%) of the participants reported that their most recent act of stalking perpetration occurred more than a year previously, and only about a quarter (23.8%) of the participants were actively engaging in stalking perpetration. Regarding lifetime stalking perpetration, 41.8% of the respondents engaged or had engaged in stalking behaviors once to several times a day, and most periods of stalking perpetration (39%) lasted 2 to 12 months. The majority of the stalkers (85%) targeted someone they knew (i.e., a non-stranger), with 45% targeting current or former intimate partners and 40% targeting non-intimate non-strangers (a friend, roommate, neighbor, relative, work or school colleague, or acquaintance).

### 3.2. Prevalence of Lifetime Stalking Perpetration Behaviors

The prevalence of different stalking behaviors perpetrated throughout the participants’ lifetimes is shown in Table 2. Surveillance-oriented stalking behaviors were found to be most frequently perpetrated (17.8–42.2%), followed by approach-oriented stalking behaviors (13.3–31.1%) and intimidation- and aggression-oriented stalking behaviors (6.7–26.7%). The following stalking behaviors were most frequently perpetrated by the participants during their lifetimes: (1) followed or spied on the victim (42.2%) and stood outside the victim’s home, school, or workplace (42.2%), (2) showed up at the victim’s location with no reason to be there (37.8%), and (3) made unsolicited phone calls to the victim (31.1%). Notably, one significant sex difference, which approached a moderate effect size, was found: 25% of females and 6.1% of males reported that they had “ever threatened to harm or kill the victim” (χ^2^ = 3.20, Phi = −0.27, *p* = 0.049). This result indicates that female participants who engaged in stalking perpetration behaviors were 5.17 (confidence interval [CI] = 0.75, 35.85) times more likely to threaten to harm or kill their victim. Male participants, however, were 5.26 (OR = 0.19; CI = 0.03, 1.34; formula = 1/[1 − P]) times less likely to threaten to harm or kill their victim.

### 3.3. Lifetime Stalking Perpetration Motives

Table 3 presents participants’ lifetime stalking perpetration motives. In general, the most frequently reported stalking perpetration motives were as follows: (1) found the victim attractive (71.1%), (2) to get the victim back into a relationship (62.2%), and (3) to control the victim (37.8%). A significant sex-related difference was found for “the victim caught me doing something” (50% females vs. 21.2% males; χ^2^ = 3.55, *p* = 0.048). The strength of this relationship approached a moderate effect size (Phi = −0.28). Female participants who engaged in stalking perpetration were 3.71 (CI = 0.91, 15.15) times more likely to do so because the victim caught them doing something. The opposite was true for male participants, as they were 3.70 (OR = 0.27; CI = 0.07, 1.10) times less likely to engaging in stalking because the victim caught them doing something.

### 3.4. Prevalence of Other Violent and Nonviolent Offending Behaviors

Table 4 demonstrates the lifetime prevalence of additional violent and nonviolent offending behaviors perpetrated against victims by the participants. In general, the most frequently reported violent and nonviolent behaviors by the participants were as follows: (1) raped/sexually assaulted the victim (17.8%), (2) attacked/attempted to attack the victim’s pet (15.6%), and (3) illegally entered the victim’s house/apartment (11.1%). Notably, “raped/sexually assaulted the victim” was found to be the most frequently reported violent behavior by both male and female participants (18.2% males and 16.7% females). Although there was no significant sex-related difference at the level of *p* < 0.05, there was one prominent sex-related difference: 8.3% of females compared to 0% of males reported having hit/slapped/knocked down the victim (*p* = 0.094).

## 4. Discussion

Two significant and meaningful sex differences emerged from this study. Significantly more female than male participants reported threatening to harm or kill their victims. This finding is consistent with earlier studies that examined differences in the nature and prevalence of stalking violence between male and female stalkers [27,35]. However, it should be noted that making a threat is different than acting on that threat [36]. Stalkers who issue a threat do not necessarily carry out the threat, although making threats is a significant risk predictor of subsequent stalker violence [20]. This is substantiated by Meloy and Boyd [21], who found a greater likelihood of violence if a threat was communicated (30% true-positive rate) by female stalkers to their victims. These researchers identified only a one in seven chance that a female stalker would attack their victim without having issued a previous threat.

Additionally, female participants were more likely than male participants to engage in stalking perpetration because they believed that their victims had witnessed them doing something illegal or suspicious. This form of stalking behavior was more frequently observed among female stalkers (50%, 3rd most common behavior) than male stalkers (21.2%, 5th most common behavior) and is possibly rooted in a desire to intimidate the victims into silence. The rate of this motivation for stalking has been reported in past non-sex-disaggregated studies as between 6% and 29%, e.g., [37,38,39]. This category of stalkers seems to fit best into Mullen and colleagues’ [40] “resentful stalker” subtype. Resentful stalkers aim to intimidate and terrorize their victims to gain a sense of power and control. Their stalking behaviors include harassing friends of the victim, engaging in cyberstalking activities, and vandalizing the victim’s home, car, and possessions. Further research is necessary to shed more light on motivations for stalking and to determine whether these motivations may be influenced by culture.

This study was not without its limitations. For example, this study was limited by its small sample size and the use of self-reported data and a lack of depth in participants’ responses regarding their perpetration experiences. Reporting biases, such as social desirability and acts of denial, may lead participants to under-report their deviant behaviors. Future work could explore additional offender characteristics in conjunction with other offending and circumstantial factors (perhaps through in-depth follow-up interviews) to acquire more comprehensive responses. This could help us to better understand the nature and severity of male and female stalker violence and threats. Future studies should also examine gender outside the male-female binary (e.g., same-sex stalking). Although the study sample was not generalizable to the entire young adult population of Hong Kong, it may be considered representative of Hong Kong’s university student population given its large size and approximately 90% response rate. Even though this study adopted mostly preexisting measures with good validity and reliability, the measures were developed in the West and therefore may not adequately capture experiences within a Chinese society (i.e., Hong Kong). Moreover, not all possible stalking behaviors and motives are listed in the measures, with only more commonly reported behaviors and motives being included. Therefore, future research may consider adopting a mixed-methods approach to data collection and developing measures that may better capture non-Western stalking perpetration experiences. Knowledge about the stalking perpetration dynamics of male and female participants may offer insights for investigative prioritization, especially when the sex of the stalker remains unknown. This study provides a starting point for further explorations of the extent and nature of gendered threats and motivations, which will preferably be based on police evidence. For now, cautious interpretation of the results is required given the small sample size and correlational nature of the analyses. Multiple factors may have influenced the decision-making of the offenders.

## 5. Conclusions

The literature has consistently demonstrated that males and females engage in different stalking perpetration behaviors and hold different motives for their actions. This study is the first empirical research into stalking perpetration behaviors and motives in Hong Kong and provides a solid groundwork for further research, regardless of its limitations. Stalking has yet to be legislated against in most non-Western countries, and this study is therefore important because it empirically demonstrates the varied nature of stalking perpetration and the effects that stalking perpetration may have on victims, regardless of geographical location. This study will hopefully encourage legislation in jurisdictions that do not currently have specific laws dealing with stalking. Recognizing stalking perpetration dynamics (e.g., offending characteristics, lifetime stalking perpetration behaviors and motives, and other violent and nonviolent behaviors) is pivotal for constructing effective intervention strategies to reduce the propensity for engaging in stalking perpetration behaviors and the escalation into more serious types of offending behavior (e.g., resulting in sexual assault and homicide, [41,42]). More specifically, because of the sex differences associated with different stalking perpetration characteristics, behaviors, and motives, it may be effective for mental health professionals and social service providers to develop new intervention protocols or to refine existing strategies to be more gender-specific.

## Figures and Tables

**Table 1:** Offending characteristics of the most recent (past 12 months) stalking perpetration by young adults in Hong Kong (*N* = 45).

Characteristics	All Sample	Male (n = 33)	Female (n = 12)	Sex Differences	
	*M* (*SD*)/*N* (%)	*M* (*SD*)/*N* (%)	*M* (*SD*)/*N* (%)	χ^2^ (df)	*t* Value/Phi/Cramer’s V
Engaged in stalking perpetration in the past 12 months (n = 43)				3.41 (1)	0.28 *
Yes	18 (41.9)	16 (50.0)	2 (18.2)		
No	25 (58.1)	16 (50.0)	9 (81.8)		
Most recent incident of stalking perpetration (n = 41)				2.12 (3)	0.23
Currently	3 (7.3)	3 (10.0)	0 (0.0)		
Within a month	12 (29.3)	9 (30.0)	3 (27.3)		
Two to 12 months ago	6 (14.6)	5 (16.7)	1 (9.1)		
More than a year ago	20 (48.8)	13 (43.3)	7 (63.6)		
Currently engaging in stalking perpetration (n = 42)				0.10 (1)	−0.05
Yes	10 (23.8)	7 (22.6)	3 (27.3)		
No	32 (76.2)	24 (77.4)	8 (72.7)		
Frequency of stalking perpetration (n = 43)				4.12 (5)	0.31
Once to several times a day	18 (41.8)	11 (34.4)	7 (63.6)		
Once a week	10 (23.2)	8 (25.0)	2 (18.2)		
Once a month	3 (7.0)	2 (6.3)	1 (9.1)		
Once in two to 12 months	3 (7.0)	3 (9.4)	0 (0.0)		
Once in several years	2 (4.7)	2 (6.3)	0 (0.0)		
Unspecified or inconsistent in frequencies	7 (16.3)	6 (18.6)	1 (9.1)		
Duration of stalking perpetration (n = 41)				1.31 (3)	0.18
Less than a month	10 (24.4)	9 (28.1)	1 (11.1)		
One month	10 (24.4)	7 (21.9)	3 (33.4)		
Two to 12 months	16 (39.0)	12 (37.5)	4 (44.4)		
More than a year	5 (12.2)	4 (12.5)	1 (11.1)		
Stalker−victim relationship (n = 40)				4.16 (2)	0.32
Intimate partner (current/former spouse/boyfriend/girlfriend)	18 (45.0)	15 (48.4)	3 (33.3)		
Non−intimate non−stranger (friend/roommate/neighbor/relative/known from school or work/acquaintance)	16 (40.0)	10 (32.3)	6 (66.7)		
Stranger	6 (15.0)	6 (19.4)	0 (0.0)		

* *p* < 0.05.

**Table 2:** Prevalence and unadjusted odds ratio of lifetime stalking perpetration behaviors of young adults in Hong Kong (N = 45).

Behaviors	Frequencies of Stalking Perpetration Behavior (%)				Male as Stalker	Female as Stalker
	All Sample	Male (n = 33)	Female (n = 12)	χ^2^ (Phi)		
	*N* (%)	*N* (%)	*N* (%)		OR (95% CI)	OR (95% CI)
Surveillance items						
1. Followed or spied on the victim	19 (42.2)	14 (42.4)	5 (41.7)	0.01 (0.01)	1.03 (0.27, 3.94)	0.97 (0.25, 3.70)
2. Showed up at places the victim was although he/she had no business being there	17 (37.8)	13 (39.4)	4 (33.3)	0.14 (0.06)	1.30 (0.32, 5.21)	0.77 (0.19, 3.08)
3. Stood outside the victim’s home, school, or workplace	19 (42.2)	16 (48.5)	3 (25.0)	1.99 (0.21)	2.82 (0.65, 12.33)	0.35 (0.08, 1.55)
4. Contacted the victim’s friends/family to learn of his/her whereabouts	8 (17.8)	5 (15.2)	3 (25.0)	0.58 (−0.11)	0.54 (0.11, 2.70)	1.87 (0.37, 9.40)
Approach items						
5. Tried to communicate with the victim against his/her will	13 (28.9)	9 (27.3)	4 (33.3)	0.16 (−0.06)	0.75 (0.18, 3.12)	1.33 (0.32, 5.54)
6. Made unsolicited phone calls to the victim	14 (31.1)	11 (33.3)	3 (25.0)	0.29 (0.08)	1.50 (0.34, 6.68)	0.67 (0.15, 2.97)
7. Sent the victim unsolicited letters or written correspondence	13 (28.9)	11 (33.3)	2 (16.7)	1.19 (0.16)	2.50 (0.47, 13.44)	0.40 (0.07, 2.15)
8. Sent unsolicited or harassing emails to the victim	6 (13.3)	5 (15.2)	1 (8.3)	0.35 (0.09)	1.96 (0.21, 18.78)	0.51 (0.05, 4.87)
Intimidation and aggression items						
9. Made the victim feel fearful for his/her safety or life	3 (6.7)	2 (6.1)	1 (8.3)	0.07 (−0.04)	0.71 (0.06, 8.62)	1.41 (0.12, 17.12)
10. Left unwanted items for the victim to find	12 (26.7)	9 (27.3)	3 (25.0)	0.02 (0.02)	1.13 (0.25, 5.12)	0.89 (0.20, 4.04)
11. Vandalized the victim’s property/destroyed something he/she loved	6 (13.3)	3 (9.1)	3 (25.0)	1.93 (−0.21)	0.30 (0.05, 1.75)	3.33 (0.57, 19.48)
12. Ever threatened to harm or kill the victim	5 (11.1)	2 (6.1)	3 (25.0)	3.20 (−0.27) *	0.19 (0.03, 1.34)	5.17 (0.75, 35.85)

Notes. Odds ratios (OR). Confidence interval (CI). Reference codes: 1 = male, 0 = female, * *p* < 0.05.

**Table 3:** Prevalence and unadjusted odds ratio of lifetime stalking perpetration motives of young adults in Hong Kong (*N* = 45).

Motives	Frequencies of Stalking Perpetration Motive (%)				Male as Stalker	Female as Stalker
	All Sample	Male (n = 33)	Female (n = 12)	χ^2^ (Phi)		
	*N* (%)	*N* (%)	*N* (%)		OR (95% CI)	OR (95% CI)
1. To retaliate against the victim	9 (20.0)	8 (24.2)	1 (8.3)	1.39 (0.18)	3.52 (0.39, 31.66)	0.28 (0.03, 2.56)
2. To control the victim	17 (37.8)	14 (42.4)	3 (25.0)	1.14 (0.16)	2.21 (0.50, 9.69)	0.45 (0.10, 1.98)
3. Due to mentally ill/emotionally unstable	6 (13.3)	4 (12.1)	2 (16.7)	0.16 (−0.06)	0.69 (0.11, 4.36)	1.45 (0.23, 9.16)
4. Found the victim attractive	32 (71.1)	23 (69.7)	9 (75.0)	0.12 (−0.05)	0.77 (0.17, 3.45)	1.30 (0.29, 5.86)
5. To keep the victim back in relationship	28 (62.2)	21 (63.6)	7 (58.3)	0.11 (0.05)	1.25 (0.32, 4.82)	0.80 (0.21, 3.08)
6. Due to substance abuse	1 (2.2)	1 (3.0)	0 (0.0)	0.37 (0.09)	N/A	N/A
7. Due to stalked liked attention	6 (13.3)	5 (15.2)	1 (8.3)	0.35 (0.09)	1.96 (0.21, 18.78)	0.51 (0.05, 4.87)
8. The victim was a convenience target	2 (4.4)	2 (6.1)	0 (0.0)	0.76 (0.13)	N/A	N/A
9. The victim caught me doing something	13 (28.9)	7 (21.2)	6 (50.0)	3.55 (−0.28) *	0.27 (0.07, 1.10)	3.71 (0.91, 15.15)
10. Due to different cultural beliefs/background	2 (4.4)	2 (6.1)	0 (0.0)	0.76 (0.13)	N/A	N/A
11. Believe the victim liked the attention	1 (2.2)	0 (0.0)	1 (8.3)	2.81 (−0.25) ^+^	N/A	N/A
12. No specific motive	3 (6.7)	3 (9.1)	0 (0.0)	1.17 (0.16)	N/A	N/A

Notes: Odds ratios (OR). Confidence interval (CI). Reference codes: 1 = male, 0 = female. There were no cases for this particular combination of variables (N/A). ^+^ *p* < 0.10, * *p* < 0.05.

**Table 4:** Prevalence of violent and nonviolent offending behavior against the same victim in addition to stalking perpetration (*N* = 45).

Behavior	Frequencies of Violent and Nonviolent Offending Behavior (%)			
	All Sample	Male (n = 33)	Female (n = 12)	χ^2^ (Phi)
	*N* (%)	*N* (%)	*N* (%)	
1. Illegally entered the victim’s house/apartment	5 (11.1)	4 (12.1)	1 (8.3)	0.13 (0.05)
2. Illegally entered the victim’s car	1 (2.2)	1 (3.0)	0 (0.0)	0.37 (0.09)
3. Hit/slapped/knocked down the victim	1 (2.2)	0 (0.0)	1 (8.3)	2.81 (0.25) ^+^
4. Choked/strangled the victim	3 (6.7)	3 (9.1)	0 (0.0)	1.17 (0.16)
5. Attacked the victim with a weapon	1 (2.2)	1 (3.0)	0 (0.0)	0.37 (0.09)
6. Raped/sexually assaulted the victim	8 (17.8)	6 (18.2)	2 (16.7)	0.01 (−0.02)
7. Attacked/attempted to attack				
(a) The victim in some other ways	1 (2.2)	1 (3.0)	0 (0.0)	0.37 (0.09)
(b) The victim’s friend/co−worker	2 (4.4)	1 (3.0)	1 (3.0)	0.58 (−0.11)
(c) The victim’s pet	7 (15.6)	6 (18.2)	1 (8.3)	0.65 (0.12)
(d) The victim’s child	1 (2.2)	1 (3.0)	0 (0.0)	0.37 (0.09)

^+^ *p* < 0.10.

## Data Availability

The data presented in this study are available on request from the corresponding author.
